# Datasets generated by shotgun sequencing of metagenomic libraries of the guajataca water reservoir

**DOI:** 10.1016/j.dib.2018.11.114

**Published:** 2018-11-27

**Authors:** Berliza M. Soriano, Laura M. Del Valle-Perez, Luis Morales-Vale, Carlos Rios-Velazquez

**Affiliations:** a Industrial Biotechnology Program University of Puerto Rico, Mayaguez, Puerto Rico; b Biology Department University of Puerto Rico, Mayaguez, Puerto Rico

## Abstract

The Guajataca Water Reservoir (GWR) was constructed for irrigation and to bring potable water to the northwestern region of Puerto Rico. The generation of DNA sequencing data from aquatic bodies (AB) using culture-independent approaches allows the investigation of the total microbial diversity as well as the potential anthropogenic impact. Metagenomic libraries were constructed for two GWR sampling sites and genomic information access through shotgun sequencing. After removing the bacterial host cell genome and the library fosmid sequences, the environmental genome was processed through Rapid Annotation using Subsystems Technology for Metagenomes (MG-RAST). The sequences consisted primarily of bacteria (95.70%), followed by viruses (2.94%), other sequences (0.28%) and eukaryote (0.09%). The most abundant species were *Enterobacter cloacae* (31%), *Enterobacter sp. 638* (20%), *Enterobacter cancerogenus* (10%) and *Escherichia coli* (11%). Furthermore, the subsystem data showed that 13% of the genes belong to carbohydrates functionality, 12% to clustering-based-subsystems and another 9% related to virulence-disease-and-defense (out of which 8% pertain to genes of antibiotic resistance and toxic compounds). This unique data input will serve as a baseline to a better understanding not only the microbial communities present in the AB, but also the microbial activities with potential application in biotechnological and biomedical fields.

**Specifications table**

**Table:** 

Subject area	*Biology*
More specific subject area	*Metagenomics*
Type of data	*Figures, FASTQ File*
How data were acquired	*Illumina MiSeq*
Data format	*Raw*
Experimental factors	*Environmental Water Sample, Metagenomic Library Construction, Shotgun Sequencing, and Analysis.*
Experimental features	*The metagenomic library from Guajataca Water reservoir was shotgun sequenced and annotated using MG-RAST.*
Data source location	*Two samples were collected from the Guajataca Water Reservoir located in Quebradillas, Puerto Rico* (N 18° 22′ 33.103″ W 66° 55′ 19.268″ −N 18° 23′ 33.799″ W 66° 55′ 20.174″).
Data accessibility	*Data are with this article. The metagenomic sequenced Data generated were annotated using MG-RAST, ID* mgm4771977.3 *and deposited in NCBI database under the BioSample accession SAMN09396994*

**Value of the data**

• This project embodies the first Guajataca Water Reservoir (GWR) metagenomic libraries dataset generation.
• These data provide the scientific community with the first microbial taxonomic profile and functional structure of GWR using metagenomics and shotgun sequencing.
• Data obtained from this data article can be used for a better understanding of the ecology, biology, and anthropogenic impact of unexplored environments such as AB.
• Moreover, these data deliver a comparison point for future metagenomics studies in other water reservoirs around the world.

## 1 Data

### 1.1 Background

**Fig. 1:**
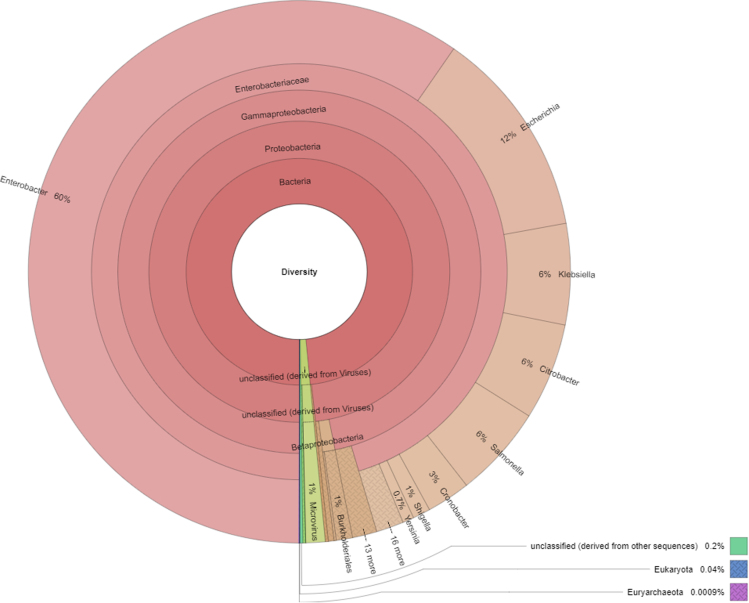
Metagenomic diversity breakdown of GWR. According to the taxonomic annotation, bacteria were the most abundant domain (95.70%) followed by viruses (2.94%), other sequences (0.28%) and eukaryotes (0.04%). However, the archaea domain was not aboundingly represented in the metagenome. A total of 18 bacterial phyla were identified from which 96.27% sequences belonged to proteobacteria follow by unclassified sequences derived from viruses (2.94%), other unclassified sequences (0.29%), *Nitrospirae* (0.13%) and unclassified sequences from bacteria (0.11%). The metagenome contained a total of 104 bacterial families where the majority belong to *Enterobacteriaceae* (93.73%; within the Gammaproteobacteria class), *Microviridae* (2.71%), *Comamonadaceae* (42%) and *Vibrionaceae* (0.39%). From a total of 182 bacterial genera, with majorities within the Enterobacteriaceae family, where Enterobacter was the most abundant with a 56.90%, followed by *Escherichia* (18.63%), *Salmonella* (7.12%), and *Klebsiella* (4.82%). Moreover, the abundant species, as per their respective genus, were *Enterobacter cloacae* (31%), *Enterobacter sp. 638* (20%), *Enterobacter cancerogenus* (10%) and *Escherichia coli* (11%). The functional structure using the NOG annotation show that a total of 78.44% of the proteins were poorly characterized, another 12.5% in information storage and processing, 6.15% of cellular processes and signaling protein and finally a 2.90% of metabolism proteins.

**Fig. 2:**
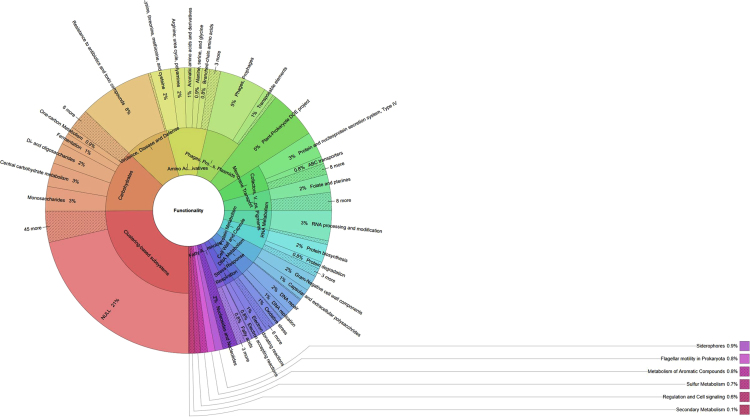
Subsystem functional structure compilation. The GWR metagenome showed that a total of 13% of the genes belong to carbohydrates, 12% to clustering-based subsystems, another 9% related to virulence, disease and defense (out of which 8% genes of resistance to antibiotics and toxic compounds), 8% amino acids and derivatives and 7% of transposable elements genes. Furthermore, the data show the presence of genes related to membrane transport (6%), cell wall and capsule (5%), and cofactor/vitamins (4%) were identified.

Among aquatic bodies (AB), water reservoirs and dams are essential because of their direct impact on humans. Water reservoirs are artificial man-made lakes to store clean water [1]. A total of 36 public reservoirs are reported to be own by the Commonwealth of Puerto Rico with 21 being considered of significant diversity and volume input [2]. The Guajataca Water Reservoir (GWR), located in Quebradillas, supplies potable water for most the northwestern region. However, its fundamental function is for agricultural irrigation for the flatlands of the area [3]. Metagenomics is a culture-independent approach that overcomes the problem of the uncultivability of most microbes to allow genomic and functional access to AB and many other environments [4]. The data presented within the data article was generated by shotgun sequencing of combined (1:1) metagenomic libraries made from two GWR samples. GWR׳s microbial diversity data are presented as a taxonomic profile (Fig. 1) and functional subsystem structure (Fig. 2).

### 1.2 Taxonomic and functional unraveling

See Figs. 1 and 2.

## 2 Experimental design, materials and methods [5], [6]

### 2.1 Metagenomic library generation

For the generation of high molecular weight metagenomic libraries, GWR water samples were collected from shallow waters (0–1 m depth). Direct DNA extraction was performed using a Metagenomic DNA Isolation Kit for Water. DNA of approximate 40 kbp was processed according to the specifications of the CopyControl™ Fosmid Library Production Kit from Epicentre. In brief, DNA was end-repaired, precipitated and ligated into a CopyControl pCC1FOS vector. MaxPlax Lambda phages were used to package the recombinant DNA and to transduce it to EPI300-T1^R^ host cells. Libraries were plated, and the number of clones determined as well as the insertion percentage by enzymatic digestion with *Bam*H1 (New England Biolabs).

### 2.2 Metagenome sequencing and pCCFOS1 vector removal

A midiprep kit (QIAGEN) was used as described by the manufacturer to extract and purify the MetaDNA from the GWR metagenomic library. The sample was then sent to the MR DNA for sequencing. Following the Qubit® dsDNA HS Assay Kit (Life Technologies) and Nextera DNA Sample Preparation Kit (Illumina) in verbatim, a genomic library was generated. DNA samples (50 ng) were diluted (2.5 ng/uL), fragmented, and finally the addition of adapter sequences. The library was diluted to 12 pM to be sequenced using a 600 cycle v3 Reagent Kit (Illumina) on the MiSeq (Illumina). To isolate the metagenome sequence, the pCCFOS1 vector was removed using the Gene Indices Sequence Cleaning and Validation script. Finally, a Blastn was performed to corroborate that no sequences from E. coli DH10B or the plasmid pCC1FOS where present in the metagenome.

### 2.3 Taxonomic and functional insights

The clean metagenomic sequences were submitted to the Rapid Annotation and subsequently examined using Subsystems Technology for Metagenomes (MG-RAST) online server to develop a functional *in silico* description of the GWR.
