# Supplementary material for: Datasets generated by shotgun sequencing of metagenomic libraries of the guajataca water reservoir
Source: Data Brief. 2018 Nov 27;21:2531–5. doi: 10.1016/j.dib.2018.11.114 (PMC6288454; doi:10.1016/j.dib.2018.11.114)
Supplement: Supplementary file 1 — Supplementary material [file mmc1.pdf]

Berliza Soriano
DIB-D-18-02007
Conflict of interest

To the Data in Brief editor,

Hereby we, the authors of the Data in Brief Manuscript No. DIB-D-18-02007, certify that the work submitted to DiB is unique and has not been published, nor it is under consideration for other journals. Furthermore, all the individuals listed as authors have approved the submission and content of the manuscript. If the accepted for publication in DiB, this manuscript will not be submitted or published into any other source without the written consent of the copyright holder.

Berliza Soriano

First Author
